# Prevalence and Factors Influencing Alcohol Use in Pregnancy among Women Attending Antenatal Care in Dodoma Region, Tanzania: A Cross-Sectional Study

**DOI:** 10.1155/2018/8580318

**Published:** 2018-10-18

**Authors:** Matunga Mpelo, Stephen Matthew Kibusi, Fabiola Moshi, Azan Nyundo, Julius Edward Ntwenya, Bonaventura C. T. Mpondo

**Affiliations:** ^1^School of Nursing and Public Health, College of Health Sciences, The University of Dodoma, Dodoma, Tanzania; ^2^School of Medicine and Dentistry, College of Health Sciences, The University of Dodoma, Dodoma, Tanzania

## Abstract

**Background:**

Alcohol use during pregnancy is high despite the well-established evidence on its adverse pregnancy outcomes and poor child development. Early identification and behavioural modification are of great significance. This study aimed to determine the prevalence and associated factors of alcohol use during pregnancy among women in Dodoma region.

**Methods:**

365 randomly selected pregnant women attending antenatal care services in Dodoma region were included. Structured questionnaires were used to assess sociodemographic characteristic and alcohol use. Both descriptive and inferential analyses were used to estimate the prevalence and independent relationships of factors associated with alcohol use in pregnancy, respectively.

**Results:**

Results showed a prevalence of 15.1% out of the 365 women attending antenatal services in Dodoma region. Prepregnancy alcohol use and having relatives who use alcohol were associated with alcohol use (AOR= 5.19; 95% CI: 4.791-34.867 and AOR=1.57; 95% CI: 1.393-6.248), respectively. Moreover, other associated factors included low education status (AOR=10.636; 95% CI: 1.89-19.844), making local brews as a source of income (AOR=11.44; 95% CI: 1.008-19.86), and not having had complications in previous pregnancies (AOR=4.93; 95% CI: 1.031-23.59).

**Conclusion:**

There is a significantly high prevalence of alcohol use during pregnancy in Dodoma. Social networks and low social, economic status were associated with alcohol use in pregnancy. There is a need for public health interventions to address alcohol use particularly targeting women of reproductive age with low socioeconomic status.

## 1. Introduction

Alcohol use during pregnancy has been confirmed for many years to be associated with negative effects on the mother and unborn child. The effect of alcohol use during pregnancy is evident immediately after birth, during early or later life particularly when it damages the Central Nervous System (CNS). Limiting the exposure to alcohol is one of the modifiable risk factors for poor pregnancy outcomes [1–4].

Binge or heavy drinking (defined as five or more drinks per occasion) is associated with Fetal Alcohol Spectrum Disorders (FASD), a more severe effect. Fetal alcohol syndrome (FAS) causes both prenatal and postnatal growth deficiency. Moreover, alcohol has been reported to be the cause of low birth rate [5–7]. Despite rampant evidence on the effects, studies show that there is an increasing trend in alcohol use during pregnancy in developing countries. In Tanzania, up to 34.1% of pregnant women have reported experiences of drinking alcohol [3].

Given the increasing prevalence of alcohol use during pregnancy, there is a need for understanding factors predisposing mothers to alcohol use during pregnancy. Previous alcohol use before pregnancy, social status, higher incomes, tobacco use, partner violence, having partners or friends who drink, late pregnancy recognition, and perceived alcohol risks had been associated with alcohol use in pregnancy. Others include traumatic history, employment, and educational status. In Tanzania availability and affordability of alcohol, maternal age, parity, religion affiliation, tribe, and HIV status contribute to high prevalence of alcohol use [8–15].

Although there is uncertainty regarding the safe level for drinking during pregnancy, research findings appear to signal that abstinence is probably the safest option. In Tanzania all antenatal care services including those located in Dodoma region recommend women to abstain from drinking during pregnancy. So this study was set to determine prevalence and factors influencing alcohol use among pregnant women in the Dodoma region.

## 2. Methods

It was a descriptive cross-sectional study which included 365 pregnant women attending antenatal care services. The study was conducted in Dodoma region the capital city of Tanzania. Dodoma region lies in the eastern-central part of the country. As per 2012 census, the region had a population of 2,083,588 [16]. It is among the least developed area of Tanzania with the highest maternal/child death rates, the majority (80%) of the Dodoma population lives in rural areas and continuously threatened by famine, and they are accustomed to engaging into drinking behaviour during agricultural off season [2]. The region is divided into seven districts, namely, Dodoma Urban, Kondoa, Mpwapwa, Chemba, Bahi, and Chamwinoand Kongwa.

### 2.1. Sample Size and Sampling Technique

A minimum sample of 332 was calculated from Cochrane formula** = Z**^2^**Pq/e**^2^, where Z is standard normal deviate, P is proportion in the target population, q is 1-p(proportion in the target population), and e is precision level.

A multiple stage sampling technique was used to select the sample. Among the seven districts, Dodoma Urban and Chamwino districts were purposively chosen due to their high population of women and to ensure representation. Then three health facilities from each district were selected purposively by considering those with more number of women in rural and urban, respectively. Subjects were selected randomly from the list of registration for the specific day.

### 2.2. Data Collection Procedure

Sociodemographic, pregnancy, and alcohol use information including age, tribe, marital status, level of education, source of income, personal hobbies, membership to the social organization, prepregnancy alcohol use, partner's alcohol use place, sharing drinking with partner, relative or relative or friend using alcohol, gestation age, pregnancy plan, pregnancy recognition, and parity status were considered as predictors of alcohol use during pregnancy and were collected using researcher's designed instrument.

Alcohol use was assessed using WHO Alcohol Use Disorders Identification Test (AUDIT). The tool is the three questions assessment tool for quick assessment of how much (quantity) and how often (frequency) an individual is drinking. Each question has the score from 0 to 4, and the total score of the tool is 12. The total score categorises the individual as low-risk drinking (0-3), moderate risk drinking (4-5), and high risk drinking (≥6).

### 2.3. Data Analysis

The analysis was done by using Statistical Product and Service Solution (SPSS) computer program version 21 which was developed by IBM-Incorporation in 2012. Descriptive statistics were summarized into frequencies and proportions. The factors associated with alcohol consumption in pregnancy were first computed by Chi-square test and variables that reached the statistically significant level of P≤0.05 were further modeled into the multiple logistic regression analysis.

## 3. Results

### 3.1. Description of the Participants

Of all 365 pregnant women with age ranging between 15 and 44 years, the mean age was 26.02 ± 6.0, with 15% of the surveyed population being below 20 years of age and 2.7% being above 40 years of age. The majority of the participants (78.9%) were married, and Gogo was the predominant tribe with (70.1%) of the participants. The majority (64.1%) of the participants' attained maximum of primary level education and 90.1% were unemployed (Table 1).

As for the reproductive profile, the majority of the participants had at least one previous pregnancy, and 81.9% were in the second trimester of the index pregnancy (Table 2)

Up to 15.1% of the participants in this study reported having consumed alcohol in the index pregnancy. Educational status (X^2^, p<0.001), the source of income (X^2^,p<0.05), type of hobbies (X^2^, p<0.001), prepregnancy alcohol use (X^2^, p<0.001), having a partner (X^2^, p<0.001), a friend (X^2^, p<0.001), or a relative (X^2^, p<0.001), sharing of drinks (X^2^, p<0.001), and preferred place for drinking (X^2^, p<0.001) with a partner and having had a history of complications in previous pregnancy (X^2^, p<0.001) were all significantly associated with alcohol consumption during pregnancy (Table 3).

Factors significantly associated with alcohol use included educational status (AOR 10.636, 95% Cl 1.890-19.844, p=0.002), making local brews as a source of income as compared with those in small business (AOR 11.444 95% Cl 1.008-19.861 p= 0.04), prepregnancy alcohol use (AOR, 5.19, Cl (4.791-34.867) p= 0.003) having relatives who use alcohol (AOR 1.57 95% Cl 1.393-6.248 p= 0.001), and not having had complications in previous pregnancies (AOR 4.93 95% Cl 1.031-23.590 p= 0.0046) (Table 4).

## 4. Discussion

This study aimed to determine the prevalence and risk factors of alcohol use among pregnant women attending antenatal care services. The findings revealed that about 15.1% of women consumed alcohol during pregnancy and this consumption was significantly associated with educational status, prepregnancy alcohol use, a source of income, having relatives drinking alcohol, and experiencing complications in the previous pregnancies.

The current study managed to establish a prevalence of 15.1% using standardized tools to assess alcohol use among pregnant women. This seems to be lower than the proportion of 21.5% reported in Northern Tanzania in year 2010 also lower than the observation from other African studies where the prevalence ranged from 30% to 59.2% [3–6] but somewhat higher than the prevalence reported in developed countries such as Sweden and Canada with prevalence of 12% and 10.8%, respectively [7, 8]. The difference on the prevalence between this study and the study from Northern Tanzania may partly be explained by the differences in methodology, as this was a cross-sectional design which interviewed women from the antenatal clinic while the Northern Tanzania study was a registry-based survey. This study has shown that higher education level is associated with alcohol use during pregnancy. This observation is consistent with results found in Ethiopia, Sweden and Japan [10–12]. However, in countries such as Spain, higher education is among the factors influencing alcohol cessation during pregnancy [13]. Although it was expected that women with higher education would be aware of the risk of drinking during pregnancy this study found a positive relationship; this gives the reason for further research to examine the association between alcohol use during pregnancy and educational status.

This study also found that prepregnancy alcohol use predicts drinking during pregnancy. This is consistent with to date literature that alcohol use before pregnancy is the best predictor of drinking during pregnancy [11, 14, 15]. Alcohol produces a physiological effect such as the strong desire to consume despite knowing the outcomes [17]; thus when an individual has drinking tendency this becomes a habit that is difficult to break [18] or one may develop withdrawal symptoms that make abstinence difficult even after conceiving. Possibly most of the women with alcohol prepregnancy do experience withdrawal symptoms that make difficult for them to stop abruptly after becoming pregnant.

Having relatives with drinking behaviour has been associated with drinking during pregnancy in this study. Thus women whose partner and relatives use alcohol are more likely to drink during pregnancy. This is consistent with findings from previous studies; one study revealed that women admitted they would be less likely to drink if their partner and relatives stopped from drinking during pregnancy period [15]. In African countries south of Sahara male partner drinking behaviour is among the risk factor for women to drink alcohol during pregnancy [19]. This might be because relatives plays a role as an essential role model for an individual to decide to drink and sometimes one can be invited to drink alcohol and becomes difficult for her to resist.

Besides, this study found that making local alcohol as the source of income increased the likelihood of consuming alcohol during pregnancy. Making and selling traditionally made alcohol increase the access since the women can take in the house which appears to be at ease place for them to drink alcohol [20]. Women who had never experienced complications in the previous pregnancy were more likely to use alcohol in the index pregnancy. However, some studies have shown that there is no association between alcohol consumption during pregnancy and previous pregnancy complications [10]. This can be explained as women who have never experienced any difficulty is easier for them to drag into risky behaviour like alcohol use.

## 5. Conclusions

The study findings revealed that alcohol use was common during pregnancy in Dodoma region. The predisposing factors for alcohol use during pregnancy were shown to be being using alcohol before pregnancy, having a relative who is using alcohol, making local brews as the source of income and experience of getting complication with alcohol use in previous pregnancies. Interventional studies are needed to come up with an effective strategy to reduce the prevalence of alcohol use during pregnancy.

## Figures and Tables

**Table 1:** Participants' distribution by sociodemographic and reproductive profile (n=365).

**Variable**	**Frequency**	**Percentage (**%**)**
***Age group***		
15-19	54	14.8
20-24	106	29
25-29	105	28.8
30-34	63	17.3
35-39	27	7.4
40+	10	2.7
***Tribe***		
Gogo	256	70.1
Rangi	26	7.1
Others	83	22.7
***Marital status***		
Single	32	8.8
Married	288	78.9
Cohabiting	45	12.3
***Educational status***		
≤ Primary education	303	83
Post-primary education	62	17
***Employment status***		
Employed	17	4.7
Unemployed	329	90.1
Self-employed	19	5.2
***Participant's hobbies***		
Making stories with friends	113	31
Watch Television/listen to Radio	150	41.1
Music (singing or dancing)	102	27.9

**Table 2:** Participants' distribution by reproductive profile (n=365).

**Variable**	**Frequency**	**Percent (**%**)**
***Parity status***		
0	106	29
1 to 2	150	41.1
≥ 3	109	29.9
***Gestation age***		
First trimester	66	18.1
Second trimester	299	81.9
***Complication in previous pregnancies***		
Yes	33	9
No	332	91
***Pregnancy plan of the participant***		
Planned	195	53.4
Unplanned	170	46.6

**Table 3:** Factors associated with alcohol use among pregnant women attending Reproductive and Child Health (RCH) clinic in Dodoma using chi-square (n=365).

**Variable**	**Ever drank in the index pregnancy**			
	**Yes**	**No**	**X2**	**P-value**
***Educational status***				
≤ Primary education	43 (14.2)	250 (85.6)		
Post-primary education	12 (19.4)	60 (80.8)	34.98	0.000
***Source of income***				
Small business	21 (13.0)	141 (87.0)		
Farming	28 (14.8)	161 (85.2)	8.2	0.045
Making local brews	6 (42.9)	8 (57.1)		
***Participant's hobbies***				
Making stories	27 (23.9)	86 (76.1)		
Television/ radio	11 (7.3)	139 (92.7)	23.238	0.000
Music (sing/ dancing	17 (16.7	85 (83.3)		
***Pre-pregnancy alcohol use (6 months)***				
Yes	47 (70.1)	20 (29.9)		
No	8 (2.7)	290 (97.3)	194.5	0.000
***Partner drinking alcohol***				
Yes	37 (28.7)	92 (71.3)		
No	18 (7.6)	218 (92.4)	26.9	0.000
***Partner drinking place***				
At home	12 (44.4)	15 (55.6)		
Outside home	25 (22.7)	85 (77.3)	28.9	0.000
never drinks	18 (7.9)	210 (92.1)		
***Drinking with husband***				
Yes	22 (61.1)	14 (38.9)		
No	33 (10.0)	296 (90.0)	66.158	0.000
***Friends using alcohol***				
Yes	40 (28.8)	99 (71.2)		
No	15 (6.6)	211 (93.4)	32.96	0.000
***Relatives using alcohol***				
Yes	46 (26.0)	131 (74.0)		
No	9 (4.8)	179 (95.2)	32.02	0.000
***Previous pregnancies complications***				
Yes	12 (36.4)	21 (63.6)		
No	43 (13.0)	289 (87.0)	12.856	0.001
***Pregnancy plan***				
Planned	22 (11.3)	17 (88.7)	4.69	0.0039
Unplanned	33 (19.4)	137 (80.6)		

**Table 4:** Factors associated with alcohol use among pregnant women attending Reproductive and Child Health (RCH) clinic in Dodoma region (n=365).

**Variable**	**OR (95**%** CL)**	**P-value**	**AOR (95**%** CL)**	**P-Value**
***Educational status***				
≤ Primary education	1		1	
Post- primary education	2.367 (1.065- 5.262)	0.000	10.636 (1.890-19.844)	0.002
***Source of income***				
Small business	1		1	
Farming	1.168 (0.635- 2.148)		2.485 (0.651-9.020)	
Making local brews	5.036 (1.589- 15.96)	0.049	11.444 (1.008- 19.86)	0.04
***Participant's hobbies***				
Music	1		1	
Making stories	1.570 (0.798- 3.089)		2.547 (0.651-9.969	
Watch Tv/listen to radio	0.396 (0.177- 0.885)	0.040	0.172 (0.031-0.945)	
***Pre-preg alcohol use (6 months)***				
No	1		1	
Yes	8.519 (3.55- 20.45)	0.000	5.19 (4.791-34.867)	0.003
***Partner use of alcohol***				
No	1		1	
Yes	4.871 (2.637- 8.997)	0.000	17.176 (0.001-526.0)	
***Partner drinking place***				
Never drinks	1		1	
At home	8.978 (3.653- 22.065)	0.000	0.012 (0.00 – 38.9614)	
Outside home	3.301 (1.712-6.365)	0.000	0.003 (0.00 -8.60196)	
***Share drinking with husband***				
No	1		1	
Yes	14.095 (6.58–30.15)	0.000	0.977 (0.202-4.729)	
***Friends using alcohol***				
No	1		1	
Yes	5.684 (2.998- 10.775)	0.000	1.389 (0.408-4.729)	
***Relatives using alcohol***				
No	1		1	
Yes	6.984 (3.302-14.771)	0.000	15.662 (1.393-6.248)	0.001
***Complications in previous pregnancies***				
Yes	1		1	
No	3.841 (1.764- 8.363)	0	4.932 (1.031-23.590)	0.0046
***Pregnancy plan***				
Planned	1		1	
Unplanned	1.894 (1.056-3.397)	0.02	0.938 (0.288-3.056)	

## Data Availability

The data used to support the findings of this study are available from the corresponding author upon request.
